# Ink-jet 3D printing as a strategy for developing bespoke non-eluting biofilm resistant medical devices

**DOI:** 10.1016/j.biomaterials.2021.121350

**Published:** 2021-12-30

**Authors:** Yinfeng He, Jeni Luckett, Belen Begines, Jean-Frédéric Dubern, Andrew L. Hook, Elisabetta Prina, Felicity R.A.J. Rose, Christopher J. Tuck, Richard J.M. Hague, Derek J. Irvine, Paul Williams, Morgan R. Alexander, Ricky D. Wildman

**Affiliations:** 1 Faculty of Engineering, University of Nottingham, University Park, Nottingham, NG7 2RD, United Kingdom; 2 National Biofilm Innovation Centre, University of Nottingham Biodiscovery Institute, School of Life Sciences, University of Nottingham, University Park, Nottingham, NG7 2RD, United Kingdom; 3 Department of Organic and Medicinal Chemistry, School of Pharmacy, University of Seville, Seville, 41012, Spain; 4 Advanced Materials and Healthcare Technologies, School of Pharmacy, University of Nottingham, NG7; 5 University of Nottingham Biodiscovery Institute, School of Pharmacy, University of Nottingham, University Park, Nottingham NG7 2RD, UK

**Keywords:** biofilms, cell instructive behaviour, medical devices, ink-jet, 3d printing

## Abstract

Chronic infection as a result of bacterial biofilm formation on implanted medical devices is a major global healthcare problem requiring new biocompatible, biofilm-resistant materials. Here we demonstrate how bespoke devices can be manufactured through ink-jet-based 3D printing using bacterial biofilm inhibiting formulations without the need for eluting antibiotics or coatings. Candidate monomers were formulated and their processability and reliability demonstrated. Formulations for *in vivo* evaluation of the 3D printed structures were selected on the basis of their *in vitro* bacterial biofilm inhibitory properties and lack of mammalian cell cytotoxicity. *In vivo* in a mouse implant infection model, *Pseudomonas aeruginosa* biofilm formation on poly-TCDMDA was reduced by ~99% when compared with medical grade silicone. Whole mouse bioluminescence imaging and tissue immunohistochemistry revealed the ability of the printed device to modulate host immune responses as well as preventing biofilm formation on the device and infection of the surrounding tissues. Since 3D printing can be used to manufacture devices for both prototyping and clinical use, the versatility of ink-jet based 3D-printing to create personalised functional medical devices is demonstrated by the biofilm resistance of both a finger joint prosthetic and a prostatic stent printed in poly-TCDMDA towards *P. aeruginosa* and *Staphylococcus aureus.*

## 1 Introduction

Infections associated with implanted medical devices such as catheters, stents and prosthetic joint replacements are responsible for significant patient morbidity and mortality^[1–2]^. They are a major complication of orthopaedic and trauma surgery and impose a significant economic burden on healthcare services worldwide. Such infections are generally chronic and caused by bacterial pathogens such as *Pseudomonas aeruginosa* and *Staphylococcus aureus* forming biofilms on implant surfaces within which bacterial cells are localized in a self-generated matrix consisting of polysaccharides, proteins, lipids and extracellular DNA. Such biofilms are highly refractory to host innate immune defences and cause persistent infections that lead to chronic inflammation, collateral damage to the surrounding tissues and implant failure^[3]^.

Biofilms are also intrinsically tolerant to antibiotics such that implant-associated infections are extremely challenging to treat ^[4–6]^ often requiring the removal of the implanted device^[7–9]^. Attempts to prevent such infections include blending antibiotics into the implant materials or through surface modification to kill infecting bacteria^[3, 10–18]^. These approaches face major challenges including coating delamination and cracking within the implant surface host tissue environment ^[16]^, localized cytotoxicity from anti-microbial coatings ^[16]^, active antimicrobial compound depletion^[17,18]^, and potential selection for anti-microbial resistance resulting from the selective pressures that antimicrobial killing strategies impose^[19]^.

Consequently, the ability to manufacture bespoke biofilm resistant devices from simple biofilm inhibiting polymers without the need for eluting antibiotic actives or coatings would offer a significant healthcare advantage ^[20–23]^. To achieve this, we used additive manufacturing/3D printing, exploiting its design freedoms to manufacture personalised devices, on demand and use novel 3D printable formulations composed of monomers that are resistant to bacterial attachment and subsequent biofilm development ^[24–26]^. We developed new ink formulations using biofilm inhibiting monomers as structural components, aiming for full compatibility with ink-jet-based 3Dprinting. Such monomers were discovered from our pre-established monomer database^[25]^ following ink-jet printability assessment^[27]^. Two candidate monomers were found, formulated and tested for ink-jet based 3D-printing processibility and reliability and, once printed, assayed for their cytotoxicity to mammalian cells and bacteria as well as their biofilm inhibiting efficacy *in vitro* and *in vivo*. The data obtained highlighted their significant potential for preventing biofilm-associated infections by *Staphylococcus aureus* and *Pseudomonas aeruginosa*^[28–30]^. Inspired by the need to prevent such medical device associated infections, we manufactured a bespoke finger joint prosthetic^[31–33]^ and a prostatic stent^[34–37]^ to exemplify the range and complexity of structures printable with this technique. We demonstrate their resistance to biofilm formation such that our study not only introduces non-fouling biomaterials for such devices, but also exploits an advanced manufacturing method for medical devices that are adaptable to individual patient needs.

## 2 Results

**Figure 1:**
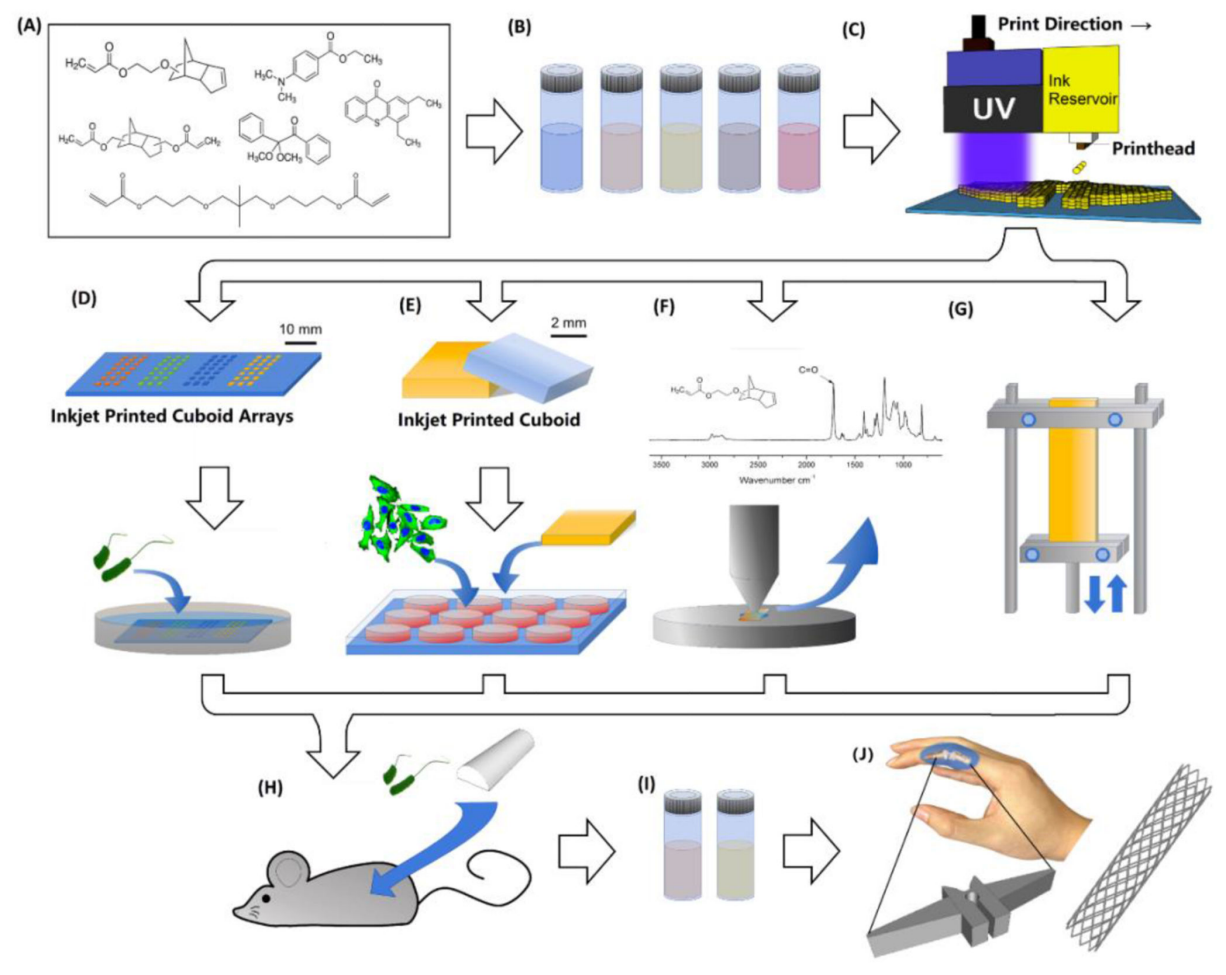
Schematic for developing optimized formulations for ink-jet based 3D-printing. ***A-B)** Monomer candidates selected for formulation development and optimization. **C)** A Fujifilm Dimatix DMP-2830 3D printer was used to print samples. The system in this case was equipped with a cartridge ejecting 10 pL drop volumes, utilising up to 16 nozzles. **D)** On-slide arrays of cuboids were created by ink-jet based 3D*-printing *for preliminary microbiology biofilm assays using* Pseudomonas aeruginosa. ***E)** Cytotoxicity and cell attachment biocompatibility tests on the printed samples were carried out using mouse embryonic fibroblast 3T3 cells to assess biocompatibility of the printed device; **F)** Attenuation Total Reflectance Infrared Spectroscopy (ATR-IR) was used to quantify the levels of residual acrylate in the specimens made from different ink formulations; **G)** Mechanical tests were performed by Dynamic Mechanical Analysis (DMA) in tension mode at room temperature; **H)** Formulations resulting in desirable properties were tested* in vivo *to ensure that the cell instructive properties were retained in a more complex environment; **I-J)** The finalized ink formulations were used to print concept devices.*

The approach taken in this study is illustrated graphically in Figure 1. Photoreactive monomer candidates were selected based on screening for resistance to bacterial biofilm formation and assessed for their capacity for consistent and reliable deposition from an ink-jet print head. Ink-jet based 3D-printing has stringent requirements on the physical and chemical properties of materials to be printed. Using printability as a guide, nine candidates (Supplementary Table S1) were explored and those outside the range commonly accepted as ‘printable’ for ink-jet^[27]^ were screened out. Trial printing of the remaining candidates was then conducted to determine the reliability of printing and whether the materials could solidify sufficiently to form 3D structures. On this basis, we found two candidates: tricyclo[5.2.1.02,6]decanedimethanol diacrylate (TCDMDA) and ethylene glycol dicyclopentenyl ether acrylate (EGDPEA). Sixteen formulations were then created, covering a range of utilities in different curing environments and potential reaction speeds (Supplementary Table S2) as it is known that these could influence final product performance^[38,39]^. Both Norrish type I (nitrogen environment) and Norrish type II (air environment) initiators were evaluated with respect to compatibility of the formulations when processing in different environments. A series of tests on each formulation was conducted to assess the performance of our 3D printed structures, including mechanical properties, *in vitro* bacterial biofilm formation and growth inhibitory effect towards bacterial cells, as well as *in vivo* assessment in a mouse infection model. Since the formulations are directed towards manufacturing of printed devices that could be used in a clinical context, their mammalian cell cytotoxicity (following ISO 10993 guidelines) and level of ink residues were also investigated. Through the analyses for biofilm coverage, mammalian cell cytotoxicity, mechanical performance and level of ink residues, a protocol for developing an ink-jet based 3D-printed functional formulation and optimized ink for meeting all the design criteria was demonstrated.

### 2.1 Mechanical properties

Dynamic mechanical analysis (DMA) measurements were carried out to determine the physical properties of the printed materials, through which it was found that the moduli of both of poly-EGDPEA and poly-TCDMDA fall into the modulus range for cancellous bone, an important consideration for bone implants^[40,41]^. The elastic moduli of poly-TCDMDA specimens were around 2.1 GPa, substantially higher than the 0.18 GPa observed for poly-EGDPEA. The mechanical performance was also found to be directly related to the photoinitiator concentration used (Supplementary Figure S1).

### 2.2 Bacterial Biofilm Formation on Polymer Cuboid Arrays

**Figure 2:**
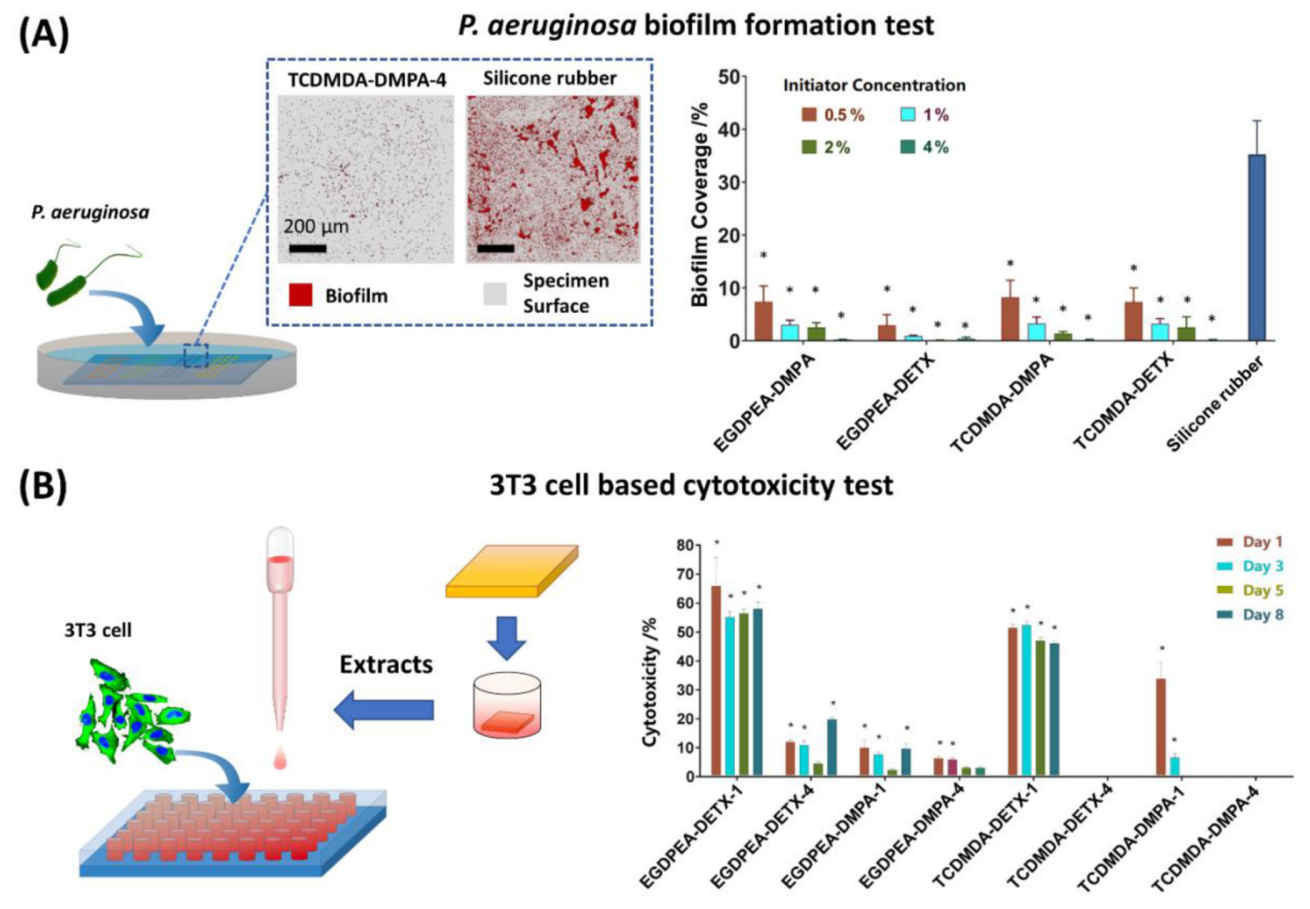
*P. aeruginosa* biofilm surface coverage and 3T3 mammalian cell based cytotoxicity assay ***A)** An array of cuboids was printed onto polystyrene slides and bacterial biofilm formation compared with a silicone control; the samples were imaged after incubation with* P. aeruginosa (tagged with the red fluorescent protein mCherry) using confocal microscopy. Biofilm formation was assessed over 640 x 640 μm and presented as biofilm coverage (%) over the whole assessment window (mean ± standard deviation, n = 24)(right); statistically significant differences (*p ≤0.001) were determined using a one-way ANOVA with post-hoc Dunnett’s test with respect to the control (right). An example confocal microscopy image of biofilm formation is included on poly-TCDMDA-DMPA-4 (left) and silicone rubber control (right). **B)** Comparison of 3T3 fibroblast cytotoxicity (%) for the printed cuboid tablets on different days, the test was performed using an LDH assay: mean ± standard deviation with n = 5; statistically significant differences (*p ≤0.05) were sought using a two-way ANOVA with post-hoc Tukey’s test with respect to the control (right); An example of the Live/Dead® cell viability assay on a poly-TCDMDA-DMPA-4 sample illustrating viable cells and proliferation (left).

To determine whether the candidates retained the desired biofilm resistance after being formulated for ink-jet based 3D-printing, they were printed using a laboratory-based ink-jet printer. For each formulation, the printed samples consisted of a series of 24 cuboid arrays (2000 x 2000 x 100 μm^3^ each) on polystyrene slides (Supplementary Figure S2). These were cultured with *P. aeruginosa* and biofilm surface coverage on each cuboid quantified after 72 h incubation. All of the printed and cured poly-EGDPEA and poly-TCDMDA surfaces showed lower biofilm surface coverage when compared with the silicone rubber control (Appleton Woods, medical grade tubing). The best performing printed poly-EGDPEA and poly-TCDMDA showed only 0.22% ± 0.04% and 0.13% ± 0.11% biofilm surface coverage compared with >30% for a silicone rubber control (Figure 2A), suggesting both materials retained their ability to prevent bacterial biofilm formation after being adapted to ink-jet based 3D-printing.

Since the reduction in biofilm surface coverage could have been caused by leaching monomer residuals, we tested the formulations at both 1% and 4% for bacterial growth inhibition. Supplementary Figure 3 shows that both *P. aeruginosa* and *S. aureus* grew to similar population densities in the presence of each of the bacterial biofilm resistant materials irrespective of whether the formulation contained 1% or 4% of the photoinitiator. Thus, the reduction in biofilm surface coverage is likely attributable to fewer monomer residuals, suggesting the ability of the material to resist biofilm development is enhanced as conversion is increased.

### 2.3 Mammalian Cell Cytotoxicity and Attachment

With respect to printed medical devices, the compatibility of the printed materials with mammalian cells must be evaluated to assure the material is non-toxic. Consequently, both cytotoxicity and cell attachment assays were carried out to assess whether printed constructs could support mammalian cell attachment and proliferation and as a first test of whether these materials could be used safely within the body. Cytotoxicity assays for mammalian cells (using 3T3 fibroblasts) were conducted using printed 5 x 5 x 1 mm^3^ cuboid samples, following the guidelines presented in ISO 10993^[42]^, the protocol is detailed in the Methods section. Based on the data in Figure 2B, only four sets of samples can be considered to have sufficiently low cytotoxicity levels rendering them as appropriate for medical device application. Conditioned media samples from poly-TCDMDA-DETX-4 and poly-TCDMDA-DMPA-4 were the only samples not to exhibit cytotoxicity at any time point, showing lactate dehydrogenase (LDH) levels similar to those of the control (Figure 2B; right hand panel). Samples from poly-EGDPEA-DMPA-4 and poly-TCDMDA-DMPA-1 showed cytotoxicity over three days, which reduced on subsequent time points. The pattern of photoinitiator content, monomer conversion (discussed in the following section) and cytotoxicity suggests that leaching of residual monomer leads to cytotoxicity, but in the case of poly-EGDPEA-DMPA-4 and poly-TCDMDA-DMPA-1, these are cleared over a timescale of 5 days. All other samples showed either no improvement over the test period, or a highly cytotoxic response indicating that these formulations would be inappropriate for clinical use. Supporting results were obtained using the complementary ‘Presto Blue’ cell viability (Supplementary Figure S4) and attachment assays (Supplementary Figure S5). These indicated that 3T3 cells attached and proliferated when cultured on poly-EGDPEA-DMPA-4, poly-TCDMDA-DETX-4, poly-TCDMDA-DMPA-1 and poly-TCDMDA-DMPA-4 surfaces. Of these, the metabolic activity of cells (determined using the Presto Blue assay) was highest on poly-TCDMDA-DETX-4 and poly-TCDMDA-DMPA-4, closely matching the trends observed for the conditioned cytotoxicity assays.

### 2.4 Spectroscopic Assessment of Curability

It was noted that the mammalian cell cytotoxicity of the device, modulus and biofilm resistance were all influenced by the level of photoinitiator concentration and therefore possibly level of conversion. To investigate this and quantify the relationships, Attenuated Total Reflectance-Infrared Spectroscopy (ATR-IR) was used to determine the residual acrylate content on the printed samples to evaluate whether there was a correlation between photoinitiator concentration, level of ink residuals and consequently, specimen performance.

Cuboids (5 x 5 x 0.2 mm^3^) were printed using the ink candidates for further acrylate residuals checks. Supplementary Figure S6 shows the acrylate residuals as a function of the photo initiator concentrations, in which the characteristic peak at 810 cm^-1^ (C-H bond out-of-plane bending vibration of the alkene group) was used to indicate the relative amount of unreacted residual alkene group (C=C). For both initiators, the concentration of the residual reduced with increasing initiator concentration, which suggests increased conversion during printing. The relationship between the level of conversion and polymer performances was assessed using the Pearson correlation coefficient (Supplementary Figure S7). For poly-EGDPEA, the Pearson correlation coefficient between residual alkene groups and mechanical performance, biofilm coverage and cytotoxicity reached 0.82, 0.69 and 0.86 respectively; while for poly-TCDMDA, these values are 0.70, 0.74 and 0.92, thereby confirming the strong link between the residual monomer quantity and key performance measurements.

### 2.5 *In Vitro* and *In vivo* Assessment of the Biofilm Resistance of the Printed Structures

**Figure 3:**
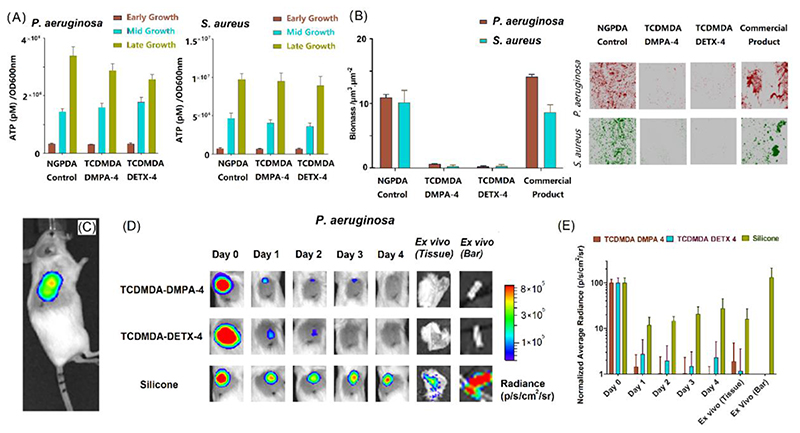
Assessment of bacterial viability and biofilm formation *in vitro* and infection *in vivo* in a mouse foreign body infection model. ***A)** Bacterial cell viability on printed specimens, RPMI-1640 medium containing the printed sample was inoculated with either* P. aeruginosa *(left) or* S. aureus *(right) cells. Intracellular ATP levels were quantified at early (OD600nm = 0.25), mid (OD600nm = 0.5) and late (OD600nm = 0.8) exponential phase using a BacTiter-Glo microbial cell viability assay, NGPDA with 4 wt% of DMPA as initiator was used as a control. Data show mean ± standard deviation, n = 3; **B)** Bacterial biofilm formation on printed specimens in vitro: the biofilm biomass of P. aeruginosa and* S. aureus *was measured after 72 h incubation. Error bars equal ± one standard deviation unit, n = 3. Fluorescent micrographs of mCherry-labelled* P. aeruginosa *(red) and GFP-labelled* S. aureus *(green) growing on each surface (right). mean ± standard deviation, n = 3. Each image is 610 x 610 μm^2^. **C)** ink-jet based 3D-printing optimized formulations (TCDMDA-DMPA-4 and TCDMDA-DETX-4) and biomedical grade silicone sections (as controls) were implanted subcutaneously in mice. After inoculation, light emission from bioluminescent P. aeruginosa at the infection site was measured on the day of inoculation. **D)** Representative bioluminescence outputs overlaid with bright field images of implanted mice infected with* P. aeruginosa *and captured on days 0 to 4. The implanted devices and surrounding tissues were also removed on day 4 from each animal and the device-associated bioluminescence quantified* ex vivo. ***E)** Bioluminescence was normalised to the output on day 0 showing that the printed devices were colonized with considerably lower levels of bacteria compared with the silicone control.*

Two ink formulations (TCDMDA-DMPA-4 and TCDMDA-DETX-4) were chosen for further assessment owing to their superior performances. Hemi-cylindrical specimens (7 mm in length and 2 mm in diameter) were printed, matching the dimensions of the control samples, and enabling sample delivery via a trocar needle in subsequent *in vivo* mouse studies. At first, the viability of *P. aeruginosa* and *S. aureus* in contact with the printed specimens was tested *in vitro* to ensure that the reduction in biofilm formation was due to colonisation resistance rather than as a growth inhibitory effect associated with either the material or photoinitiator. These experiments revealed no loss of bacterial cell viability (as quantified via intracellular adenosine triphosphate (ATP) levels) during growth in the presence of the candidate samples (Figure 3A) nor on a printed neopentyl glycol propoxylate diacrylate (NGPDA) control that promotes biofilm formation^[25]^. Since no reduction in bacterial viability was observed, it can be concluded that the material itself or any potential residuals in printed poly-TCDMDA-DMPA-4 and poly-TCDMDA-DETX-4, were not responsible for the lack of biofilm formation (Figure 3B). Planktonic bacterial growth experiments (Supplementary Figure S3) were consistent with the ATP assays and comparable with those in the presence of the NGPDA control.

Quantification of biofilm biomass and the corresponding confocal microscope images are shown in Figure 3B, which demonstrate the considerable reduction in biofilm biomass observed for both pathogens on the poly-TCDMDA-DMPA-4 and poly-TCDMDA-DETX-4 compared with the poly-NGPDA control device as well as against a sample from a commercial silicone rubber finger joint product (OSTF-0, size 0, Osteotec Ltd.).

To further understand the printed device’s performance in the much more complex host environment, *in vivo* infection experiments were carried out using a murine subcutaneous foreign body implant infection model (Figure 3C). After 4 days of post-surgical recovery, mice were inoculated with a bioluminescent strain of *P. aeruginosa* and the live infected animals imaged daily over another 5 days (day 5 to day 9 after implanting Figure 3D and 3E), a period over which infection establishes as a consequence of *P. aeruginosa* colonizing the implanted device. Light emission from the bioluminescent pathogen demonstrated the presence of metabolically active bacteria at the infection site for all samples at bacterial inoculation day 0 (Figure 3D and 3E). In contrast to the sustained light output indicative of bacterial colonization of the silicone implant, both poly-TCDMDA-DMPA-4 and poly-TCDMDA-DETX-4 showed little bioluminescence (>3 orders of magnitude reduction) consistent with resistance to bacterial biofilm formation *in vivo*. This finding was confirmed by *ex vivo* analysis of the implants and the tissues surrounding the implants after their removal from the mice and re-imaging (Figure 3D and 3E). In contrast to the TCDMDA formulations, the silicone rubber control (Clinical grade, Smith Medical) showed significantly higher bioluminescence consistent with the presence of bacterial biofilm that can also act as a reservoir for sustaining infection within the interstitial tissues surrounding the implant.

In addition, qualitative imaging of the implants using immunohistochemical staining with antibodies raised against *P. aeruginosa* cells and with the fluorescent dye, FM1-43 (as a marker for host cell and bacterial membranes) revealed evidence of a robust host response and the presence of *P. aeruginosa* cells on both poly-TCDMDA-DMPA-4 and poly-TCDMDA-DETX-4 (Supplementary Figure S8). Given the lack of bioluminescence from such samples, these bacteria are dead, killed via host antibacterial defences since the TCDMDA formulations *per se* are not bactericidal (Figure 3A and Figure S3). In contrast, the host defences were unable to kill/clear the *Pseudomonas aeruginosa* biofilm colonizing the silicone implant given the *in vivo* bioluminescence and *ex vivo* antibody labelling of the bacterial cells (Supplementary Figure S8).

**Figure 4:**
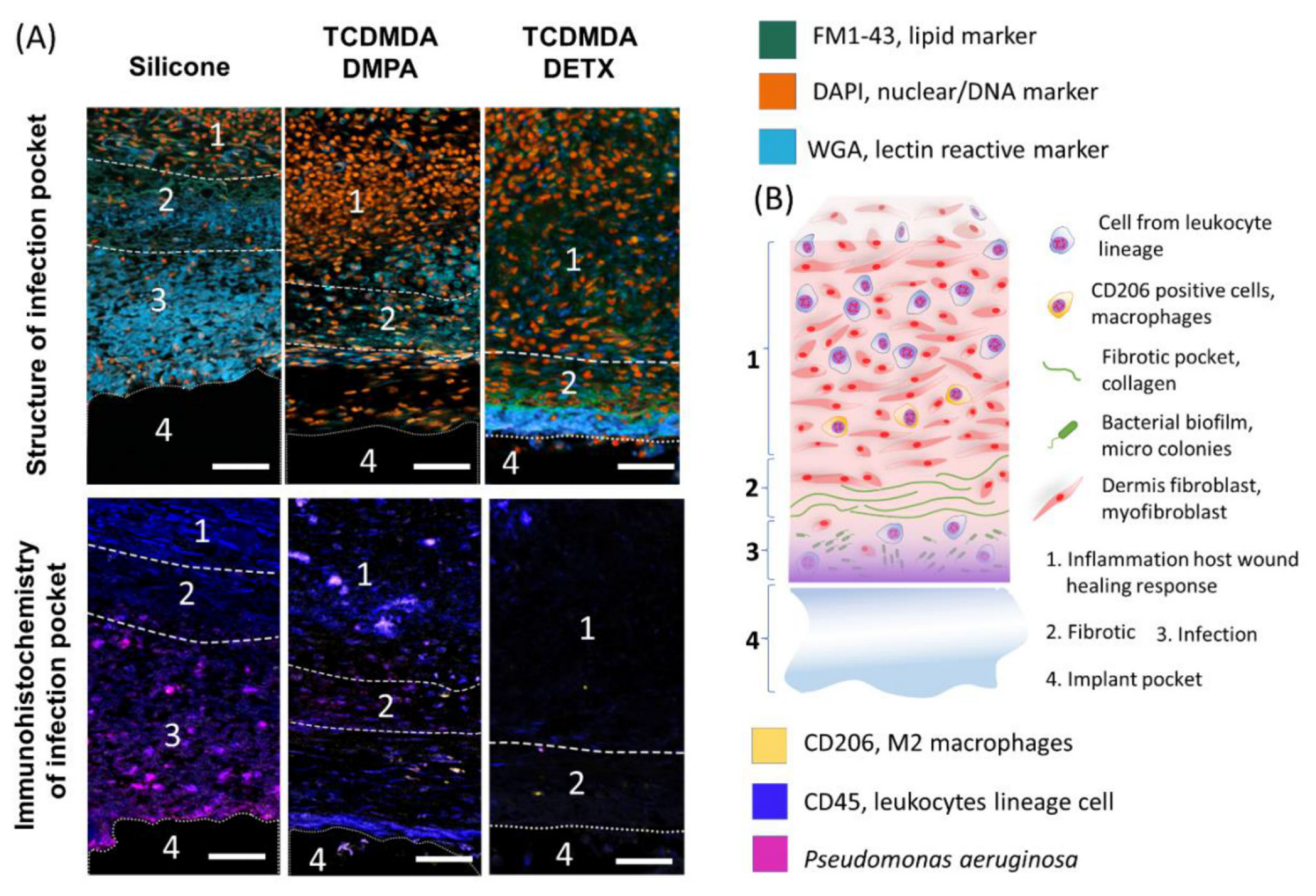
Structural assessment of the infection site and cellular localisation in tissue surrounding the implant: silicone control, TCDMDA-DMPA and TCDMDA-DETX ***A)** Structural comparison of architectural changes in tissue surrounding the implant (upper). FM1-43 membrane lipid marker (green), DAPI, nuclear/DNA (orange) and wheat germ agglutinin reactive lectin marker (cyan) staining bacterial microcolonies and the infection site; Immunohistochemical localisation (lower) of* P. aeruginosa *(magenta), CD45 leukocyte lineage cell populations (blue) and CD206 M2 macrophages (yellow), scale bar: 50 μm; **B)** schematic of the distribution of different cells in the tissue surrounding the implant.*

Further investigation of the tissue by additional staining using wheat germ agglutinin and antibodies to CD206 and CD45 markers allowed the identification of active *P. aeruginosa* infection sites and the host immune response (Figure 4). Evidence for infection/bacterial micro-colonies was observed within the silicone control group, with the infection located between the fibrotic pocket and the implant (see region 3 in Figure 4A). Conversely the two printed poly-TCDMDA implants show fewer *P. aeruginosa* micro-colonies in the surrounding tissue. Cells staining for the CD206 mannose receptor marker were observed in the surrounding tissue of both poly-TCDMDA groups, which indicate that the host is responding differently from the silicone control group. For TCDMDA-DETX, in addition to micro-colony suppression, a strong WGA response was observed suggesting that fibrotic/tissue remodeling was occurring.

The immunohistochemical staining strongly indicates that ink-jet based 3D-printed poly-TCDMDA implants are able to both provide the means to resist initial biofilm formation, and further, allow for better post-implant healing and post-infection control that does not otherwise occur when using materials such as silicone.

### 2.6 Exemplars of Ink-jet based 3D Printed Biofilm Resistant Devices

**Figure 5:**
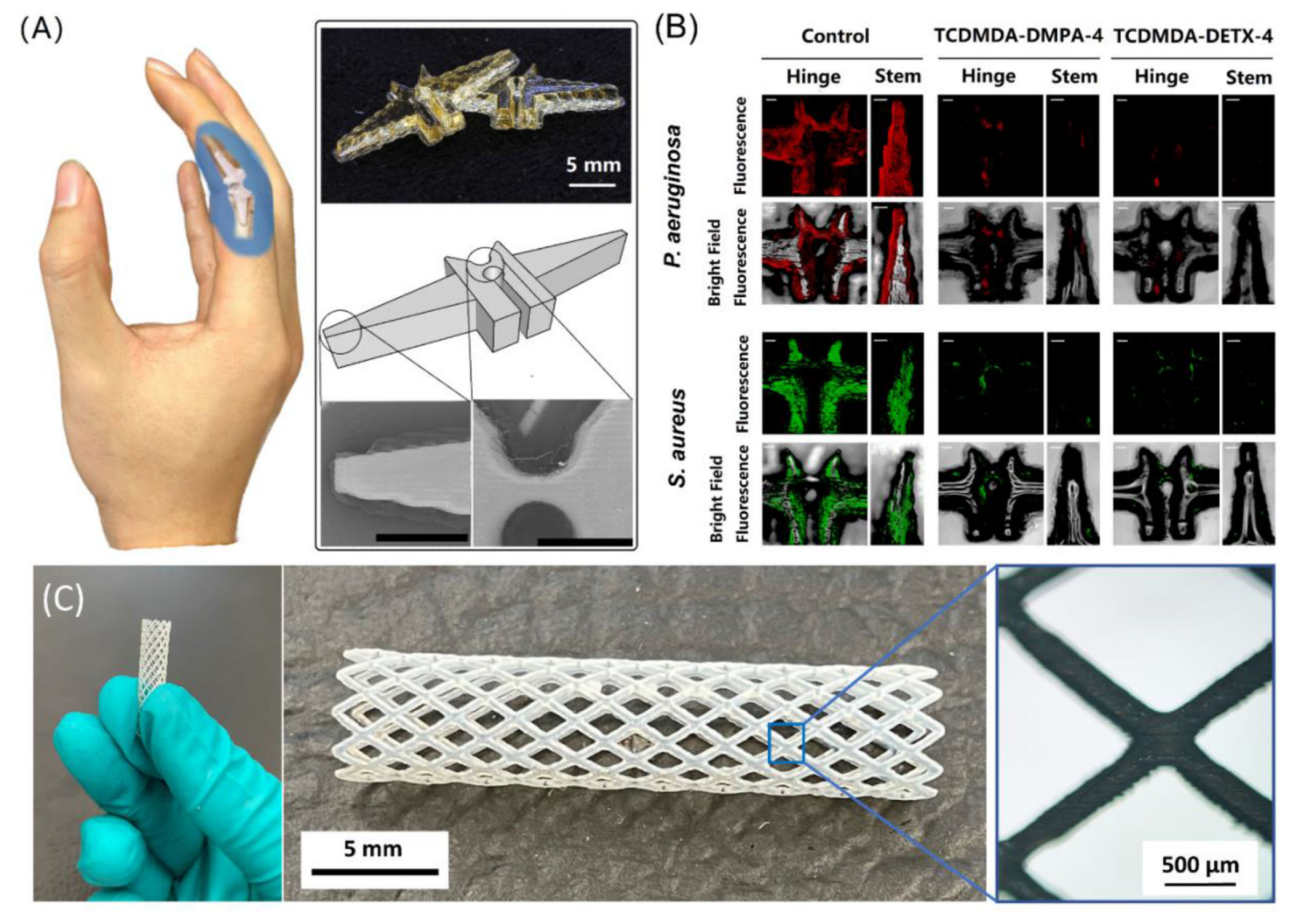
Ink-jet based 3D-printed finger prosthesis and other demonstrators using the developed ink formulations ***A)** ink-jet based 3D printed finger prosthesis with TCDMDA-DMPA-4, composed of a central hinge region between two stems, scale bars in the SEM images are 2 mm; **B)** Fluorescence and overlaid fluorescence-brightfield confocal microscopy 3D images showing in vitro biofilm formation imaged using mCherry-labelled* P. aeruginosa *(red) and GFP-labelled* S. aureus *(green) on ink-jet based 3Dprinted finger implants with the developed ink formulations. Scale bars represent 200 μm; **C)** ink-jet based 3D-printed prostatic stent exemplar with TCDMDA-DMPA-4.*

To demonstrate that an ink-jet based 3D printed functional device could be manufactured, a biofilm resistant finger joint prosthetic was firstly chosen as an exemplar (Figure 5A). Finger joint prosthetics were printed 1:1 relative to a commercial product using the best performing ink formulations (TCDMDA-DMPA-4 and TCDMDA-DETX-4). The platform used was identical to that used to produce arrays and cuboids, with a typical manufacturing time of around 4 h. The dimensions of the printed device were determined from the SEM images and compared with the CAD design (Supplementary Figures S9 and S10), showing good manufacturing accuracy. Optimisation of the manufacturing process is needed to ensure such devices could be taken forward for human use. Here we demonstrate that such a route is viable and reliable from the manufacturing perspective. Samples 1/10 of the original prosthetic dimensions were printed and tested *in vitro,* since rescaling allowed biomass assessment under full view when using a fluorescent confocal microscope; these smaller versions also showed that biofilm formation by both *P. aeruginosa* and *S. aureus* was inhibited (Figure 5B) regardless of sample geometries. To illustrate the range of complexity achievable with these formulations, a prostatic stent structure was also manufactured (Figure 5C). This lattice like structure demonstrates that complex objects with overhangs and more intricate features can be fabricated – in this case we employed a dissolvable support material to maintain the integrity of the structure during manufacture.

## 3 Conclusions

This work demonstrates that advanced cell instructive properties may be incorporated into ink-jet based 3D-printing for the production of bespoke functional medical devices. Our comprehensive set of *in vitro* and *in vivo* tests confirm the key biofilm resistance property is retained throughout our re-formulation and manufacturing process. Interestingly, our analysis reveals that our selected materials play an important role in recruiting host defences capable of clearing the infecting bacteria and preventing biofilm development. Whilst in this case we focused on the creation of devices that reduced the likelihood of infection while avoiding the opportunity for increasing antimicrobial resistance, our protocol is agnostic with respect to the cell-instructive functionality - this method offers a flexible manufacturing platform for the production of personalisable medical devices and a pathway for translation into clinical practice.

## 4 Experimental Section

### Ink preparation

All chemicals were purchased from Sigma-Aldrich and used as received. Tricyclo[5.2.1.02,6]decanedimethanol diacrylate (TCDMDA) and Ethylene glycol dicyclopentenyl ether acrylate (EGDPEA) were used as the base monomer in the preparation of all the ink formulations. The photoinitiators used were 2,2-Dimethoxy-2-phenylacetophenone 99% (DMPA) (a type I photoinitiator for nitrogen atmosphere printing) and (2,4-Diethyl-9H-thioxanthen-9-one(DETX), 98%)/(Ethyl 4-(dimethylamino)benzoate(EDB), 99wt%) (a type II photoinitiator system suitable for printing within an air atmosphere). 5 mL of each selected monomer was placed into capped vials (wrapped with aluminum foil) together with a photoinitiator (0.5 wt%, 1 wt%, 2 wt% and 4 wt%) and stirred at 800 rpm at room temperature until the photoinitiator was fully dissolved. The mixture was degassed by purging with nitrogen for 15 min to remove dissolved oxygen. The inks were filtered through a 0.45 μm filter (Minisart, Sartorius Stedim Biotech) in a dark room to remove particulates which may block printer nozzles (Figure S11). In order to maximise printability, inks were sealed and stored at 4°C overnight to help release any bubbles generated during preparation^[43]^.

### Sample printing

All the printing except the prostatic stent exemplar was carried out using a Dimatix DMP-2830. 2 mL of ink was injected into a 10 pL drop volume Dimatix cartridge containing 16 nozzles (21 μm nozzle size). The injection procedure was carried out in a dark room to prevent light-dependent inducing curing. The print cartridge was wrapped in foil to prevent ambient light curing during printing. Curing was achieved using a UV unit (365 nm and 600 mW/cm2) mounted directly on the printer allowing it to move with the printhead and induce real-time UV illumination and curing contemporaneously with deposition of material.

All the samples with DMPA as a photoinitiator were printed in nitrogen where oxygen levels were controlled to 1% ± 0.5%. The inks with DETX/EDB as initiator were printed in air.

The prostatic stent exemplar was printed with Pixdro LP50 dual head ink-jet printer with two Fujifilm Spectra SL-128 printheads. The printing was carried out in nitrogen environment with an oxygen level between 0.1% and 0.3%. Curing was achieved using a Firefly UV unit (1.5W/cm^2^@365nm). The ink was co-printed with commercial water-soluble support ink from Stratasys (SUP-707). To remove the support material. the printed structure was placed in 100 mL of deionized water for 40 minutes during which the water was replaced at 10 minutes and 20 minutes.

### Polymer mechanical and chemical properties

Dynamic Mechanical Analysis (DMA) tests were carried out at room temperature using a Perkin Elmer DMA 8000 in tension mode. Specimens were printed following a rectangular pattern (20 mm in length and 5 mm in width) with 100 layers. The test length was set to 10 mm and the width and thickness of each sample was measured prior to calculating its modulus. The test period was set to 10 min with 1 Hz extension frequency at room temperature. Infrared Spectroscopy (IR) with an ATR (Perkin Elmer UATR IR) sampling attachment was used to characterize the curability of the printed samples. The spectra for each set of samples were normalized with a peak at 1726 cm^-1^ representing the acrylate carboxyl group. The peak at 810 cm^-1^, which represents the carbon-hydrogen covalent bond on the C=C pairing was used to compare the level of conversion of the printed samples.

### Mammalian Cell Cytotoxicity

Following ISO 10993, Medical Device Tests guidance direct contact (cell attachment test) and indirect extractable testing (cytotoxicity test) was undertaken.

*Cytotoxicity test:* Samples were placed in a 96 well plate, and 1 mL of Industrial Methylated Spirit (IMS, 70% v/v, Fisher Scientific, UK) was added and allowed to evaporate overnight in a microbiological safety cabinet at room temperature. Samples were washed three times for 5 min each with PBS. Cell culture medium was added (200 μL) to each sample and kept in an incubator at 5% CO2 in air, 37°C. Conditioned medium was collected after 1, 3, 5 and 8 days, and replaced with 200 μL of fresh medium. Cell culture media were prepared by adding 10% (v/v) of Foetal Bovine Serum (FBS, Sigma-Aldrich, UK), 2 mM L-glutamine (Sigma-Aldrich, UK) and 100 U/mL penicillin, 0.1 mg/mL streptomycin and 0.25 μg/mL amphotericin B (Sigma-Aldrich, UK). Immortalized NIH 3T3 mouse embryonic fibroblast cells (3T3s, passage 60) were seeded in a 96 well plate at a density of 5000 cells/well (100 μL) and when they reached confluency, conditioned media were added and cells incubated for a further 24 hours at 5% CO2 in air at 37°C. Cells cultured in fresh media were included as a control. The lactate dehydrogenase assay (LDH Assay Kit®, Thermo Scientific) and Presto Blue® assay (Invitrogen) were used to test the cytotoxicity of the conditioned media and cell viability, respectively. The LDH assay was performed according to the manufacturer’s protocol. Two controls were performed to obtain a spontaneous and a maximum LDH activity. The spontaneous activity (Spontaneous LDH activity) was quantified using the medium collected from the controls, where cells were grown in fresh medium. To induce maximum activity (Maximum LDH activity, 100% control), 10 μL of Lysis Buffer (10X) were added to the cells grown in fresh medium for 30 min before assaying.

In brief, 50 μL of each conditioned media sample were transferred to a 96 well plate and 50 μL of the reaction mixture added to each sample, and the plate incubated at room temperature. After 30 min, 50 μL of stop solution was added. The LDH activity was measured by reading the absorbance of the samples at 490 nm (subtracted from the 680 nm reading) using a spectrofluorometer (Tecan Infinite M200 microplate reader). The cytotoxicity of the extracts was calculated using the following equation:

$$\text{\%}\;\text{Cytotoxicity}\;=\frac{\;\text{LDH}\;\text{activity}\;\text{of}\;\text{the}\;\text{sample}\;-\;\text{Spontaneous}\;\text{LDH}\;\text{activity}\;}{\;\text{Maximum}\;\text{LDH}\;\text{activity}\;-\;\text{Spontaneous}\;\text{LDH}\;\text{activity}\;}\times 100$$

*Mammalian cell metabolic activity:* After the aspiration of the medium and washing the cells in PBS, cell culture medium containing diluted Presto blue™ (1:10) was added. The samples were incubated for 45 min in an incubator at 37°C and with 5% CO2. The fluorescence intensity of the solution, which is proportional to cellular metabolic activity, was measured at 560 and 590 nm, corresponding to the excitation and emission wavelengths, respectively, and the blank reading (medium without cells) subtracted from each value.

*Mammalian cell attachment:* Samples were placed in a 48 well plate, 1 mL of Industrial Methylated Spirit (IMS, 70% v/v, Fisher Scientific, UK) was added and allowed to evaporate overnight in a microbiological safety cabinet at room temperature. Samples were washed three times for 5 min with PBS. To each sample, 400 μL of cell culture medium was added for 24 hours. 3T3 mouse fibroblast cells were seeded on the samples at a concentration of 40,000 cells/well in a total volume of 0.5 mL. After 24 hours, the materials were transferred to a new plate to measure the metabolic activity of the cells attached to the scaffold using the Presto Blue assay. Fluorescence intensity was measured and the blank (medium without cells) was subtracted from each value. The test was performed after the cells had been in contact with the test material for 1, 3, 5 and 7 days.

*Live/Dead® cell viability assay:* Calcein AM (2.5 μM, representing live cells) and ethidium homodimer-1 (5 μM, red, representing dead cells) were added to the samples and incubated for 30 min at 37°C at 5% CO2, before imaging.

Statistical analyses were performed using Prism 6 (GraphPad Software, v6.01). Two-way ANOVA was performed on cell viability followed by Tukey post-hoc test (n = 3) and on LDH results followed by the Dunnett test (n = 5). A value of p ≤ 0.05 was considered significant. For each condition, mean ± standard deviation was reported.

### Bacterial strains, growth conditions and intracellular ATP assay

*P. aeruginosa* PAO1 (Washington sub-line) and *S. aureus* SH1000 ^[42]^ were routinely grown at 37°C in LB with shaking at 200 rpm or on LB agar (2% w/v). Where required, plasmids for constitutively expressing fluorescent proteins GFP (pBK-miniTn7-egfp) and mCherry (pMMR) were introduced into the relevant host strain by conjugation or electroporation and maintained by supplementing the growth medium with the appropriate antibiotic.

For the quantification of ATP, *P. aeruginosa* and *S. aureus* cell culture samples were taken at early (OD600nm = 0.25), mid (OD600nm = 0.5) or late (OD600nm = 0.8) exponential growth phase. ATP levels were assayed using a BacTiter-GloTM Microbial Cell Viability Assay (Promega UK, Southampton, UK) according to manufacturer’s instructions.

### Bacterial Biofilm Formation

Bacterial biofilm formation assays were conducted following an established method from our previous study[25]. Briefly, the printed and UV-sterilized devices (cuboids, tablets or finger implants) with the developed formulations were inoculated with bacteria (*P. aeruginosa* tagged with the fluorescent protein mCherry, OD_600_ = 0.01) and incubated at 37°C with shaking at 60 rpm shaking for 72 h in RPMI-1640. Samples were washed 3 times with 60 rpm shaking in PBS to remove planktonic and weakly adhered bacteria before being allowed to air dry. Samples were examined using a Carl Zeiss LSM 700 laser scanning confocal microscope fitted with 405 nm, 488 nm and 555 nm excitation lasers and a 10X/NA 0.3 objective. Images were acquired using ZEN 2009 imaging software (Carl Zeiss). Bacterial surface coverage was quantified using ImageJ 1.44 software (National Institutes of Health, USA) and Comstat 2^[44]^.

### Mouse foreign body infection model

All animal experiments were approved following local ethical review at the University of Nottingham and performed under Home Office licence 30/3082. Female BALB/c mice, 19-22g (Charles River; 3 mice per infected implant and 2 mice per uninfected implant control) were housed in individually vented cages under a 12 h light cycle, with food and water *ad libitum*. The bioluminescent *P. aeruginosa* strain PAO1-L CTX: *tac-lux* was grown overnight in LB broth at 37°C, diluted 1:100 in LB and grown at 37°C to mid-log phase (OD600). The cultures were washed in PBS+10 % v/v glycerol and aliquots stored at -80°C. When required, aliquots were removed, serially diluted and cultured on LB agar plates and the number of colony forming units (CFUs) determined. One hour before device implantation via a trocar needle, Carprofen (2.5mg/kg) was administered by subcutaneous injection to reduce pain and inflammation. Animals were anaesthetised with 2% isoflurane, their flanks shaved and the skin cleaned with Hydrex surgical scrub. A small incision was made and the catheter implanted via a 9g trocar needle and closed with Gluture skin glue (Abbott Laboratories). Mice were allowed to recover for 4 days. Under anaesthesia, 10^5^ bioluminescent *P. aeruginosa* cells in 20 μL PBS were injected into printed devices implanted in the mice. The progress of bacterial infection was imaged as bioluminescence using an IVIS™ Spectrum (Perkin Elmer). The infected animals were tracked daily for 5 days via whole animal imaging for the presence of metabolically active bacteria at the infection site. After sacrificing the mice, the printed devices and the surrounding tissues were removed and re-imaged *ex vivo* using an IVIS™ Spectrum to quantify bacterial bioluminescence. In addition, the implants were fixed with 10% formal saline and subjected to immunohistochemical analysis and confocal microscopy using rabbit antibodies raised against *P. aeruginosa* cells (Invitrogen PA1-73116) and detected using a secondary goat anti-rabbit fluorescent conjugate (quantum dot 705; Thermofisher). Total host cell and bacterial membrane biomass on the implants was stained using the fluorescent cell membrane probe, FM1-43 (Thermofisher). After staining implants were imaged using by confocal fluorescence microscopy (Zeiss LSM700).

Tissues excised from the infection site and the 3D rod support were dissected out and fixed in 10% formal saline for 24 h. They were processed for paraffin embedding, from which 8 μm tissue sections were collected and dewaxed in xylene. The tissue sections were rehydrated and stained for localisation of glycoproteins using Wheat Germ agglutin Alexa 680 (Thermo Fisher), 5 μg/ml (incubation 37°C 1 h) and evaluated for tissue morphology cellular content using lipid stain FM1-43, (5 μg/ml) and total host cellular localisation using DAPI (stains intracellular DNA) incubated for 10 min at room temperature. The tissue was mounted with Fluromount (Sigma-Aldrich). Images were acquired using on a confocal fluorescence microscope (Zeiss LSM700).

Parallel 8 μm dehydrated tissue sections were rehydrated and assessed for phenotypic biomarkers. Epitope retrieval was carried out by incubation with 10 μg/ml trypsin, at 37°C for 10 min. Samples were washed three times in PBS. Tissue sections were pre-blocked at 37°C using 5% v/v bovine serum for 1 h and incubated with a primary rabbit antibody to *P. aeruginosa* (Invitrogen PA1-73116) diluted 1:500 for 2 h at 37°C. After washing 3 times in PBS tissue sections were incubated for 2 h at 37°C with a secondary anti-rabbit Alexa555 antibody, and primary antibodies to CD45 (TONBO 30-F11) eViolet405 1:50 and CD206 (Bio-Rad MCA2235A647) Alexa 647 1:50. The sections were washed 3 times in PBS and the slides mounted with Fluro mount (Sigma Aldrich). Images were acquired using a confocal fluorescence microscope (Zeiss LSM700).

## Supplementary Material

SI
