# Supplementary material for: Ink-jet 3D printing as a strategy for developing bespoke non-eluting biofilm resistant medical devices
Source: Biomaterials. Author manuscript; Available in PMC 2022 Aug 29. (PMC7613459; doi:10.1016/j.biomaterials.2021.121350)
Supplement: SI [file EMS151776-supplement-SI.docx]

Supporting Information

**Ink-jet 3D printing as a strategy for developing bespoke non-eluting biofilm resistant medical devices**

*Yinfeng He^1^† , Jeni Luckett^4^, Belen Begines^2^ , Jean-Frédéric Dubern^4^, Andrew L. Hook^3^, Elisabetta Prina^3^, Felicity R.A.J. Rose^3^, Christopher J. Tuck^1^, Richard J.M. Hague^1^, Derek J. Irvine^1^, Paul Williams^4^, Morgan R. Alexander^3*^ and Ricky D. Wildman^1*^*

Table S1: Monomers resistant to bacterial biofilm resistance after polymerization were selected from a polymer library and tested using a Dimatix DMP 2830 printing platform to assess if printable and curable using the ink-jet based 3D-printing process. Printability is assumed within the range 1 < Z < 10 [27] and an assessment of whether reliable and consistent printing was possible made by manual observation of droplet formation and deposition. Curing was assessed by observing whether a 3D printed material was self-supporting.

| Monomer | Viscosity at 25°C (cp) | Surface tension at 25°C (mN/m) | Density at 25°C (g/mL) | Z value | Printability | Cured following printing  (in air) |
| --- | --- | --- | --- | --- | --- | --- |
| Ethylene glycol dicyclopentenenyl Ether acrylate | 17.2 | 36.5 | 1.085* | 1.68 | √ | √ |
| Bisphenol A glycerolate diacrylate | >2000* | N/A | 1.18 * | N/A | × | N/A |
| Tert-butylcyclohexylacrylate | 8.8 | 44.61 | 1.108 * | 3.66 | √ | × |
| Cyclohexyl methacrylate | 2.3 | 30.5 | 0.964 * | 10.80 | √ | × |
| Tert-butyl acrylate | 0.9 | 25.1 | 0.883* | 23.97 | Not stable | × |
| Tricyclo[5.2.1.02,6]decanedimethanol diacrylate | 118.3 | 35.8 | 1.1 * | 0.24 | √ | √ |
| Neopentyl glycol propoxylate diacrylate (positive control) | 18.1 | 30.2 | 1.003 * | 1.39 | √ | √ |
| Ethyl methacrylate | 0.7 | 36.2 | 0.913 * | 37.63 | Not stable | × |
| Ethylhexyl acrylate | 1.5 | 25.9 | 0.885 * | 14.62 | Not stable | × |

*data from Sigma-Aldrich

Table S2: The composition of ink formulations and their abbreviations: Tricyclo[5.2.1.02,6]decanedimethanol diacrylate (TCDMDA), Ethylene glycol dicyclopentenyl ether acrylate (EGDPEA), 2,2-Dimethoxy-2-phenylacetophenone (DMPA), (2,4-Diethyl-9H-thioxanthen-9-one(DETX), Ethyl 4-(dimethylamino)benzoate(EDB), neopentyl glycol diacrylate (NGPDA)


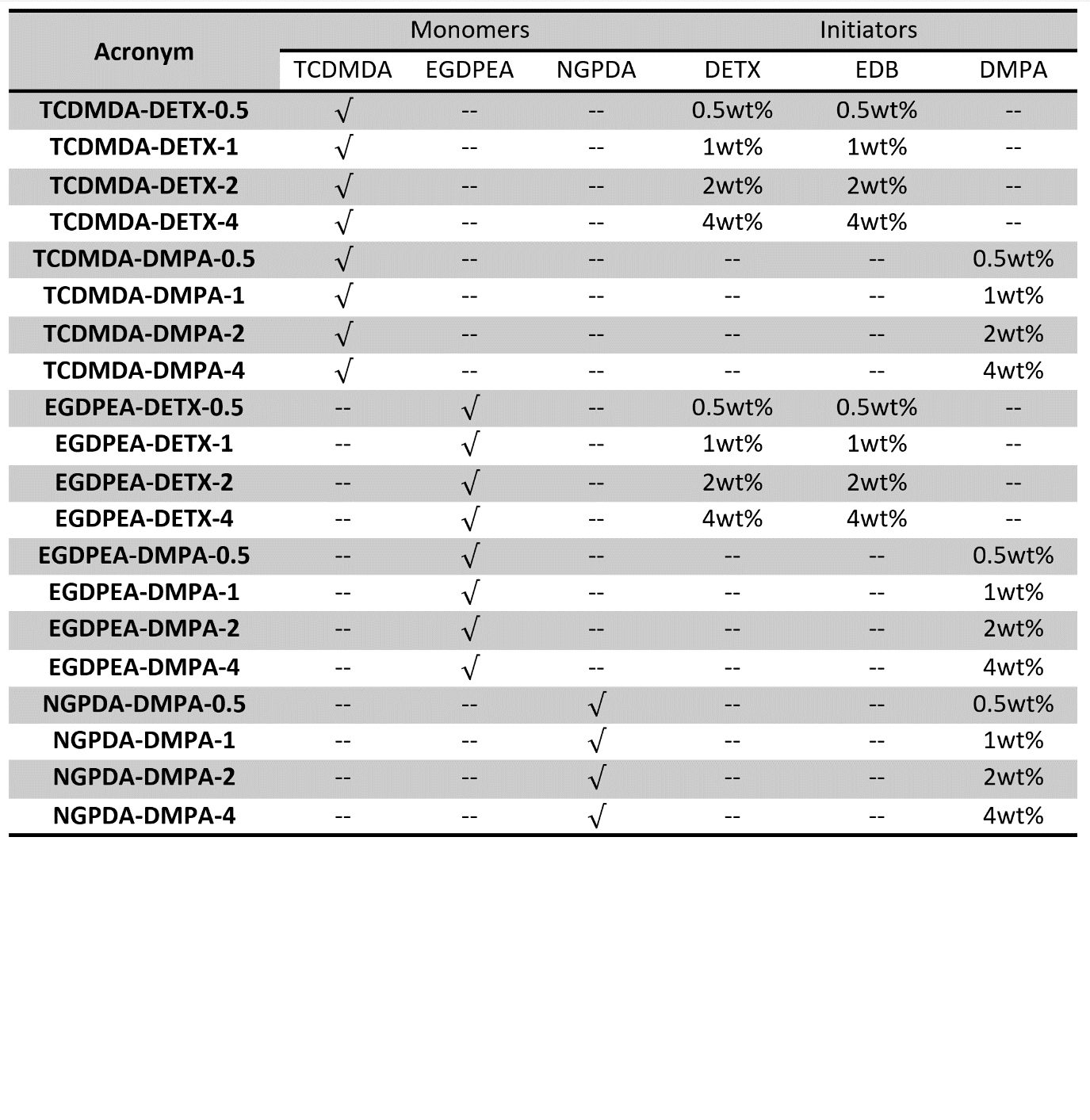


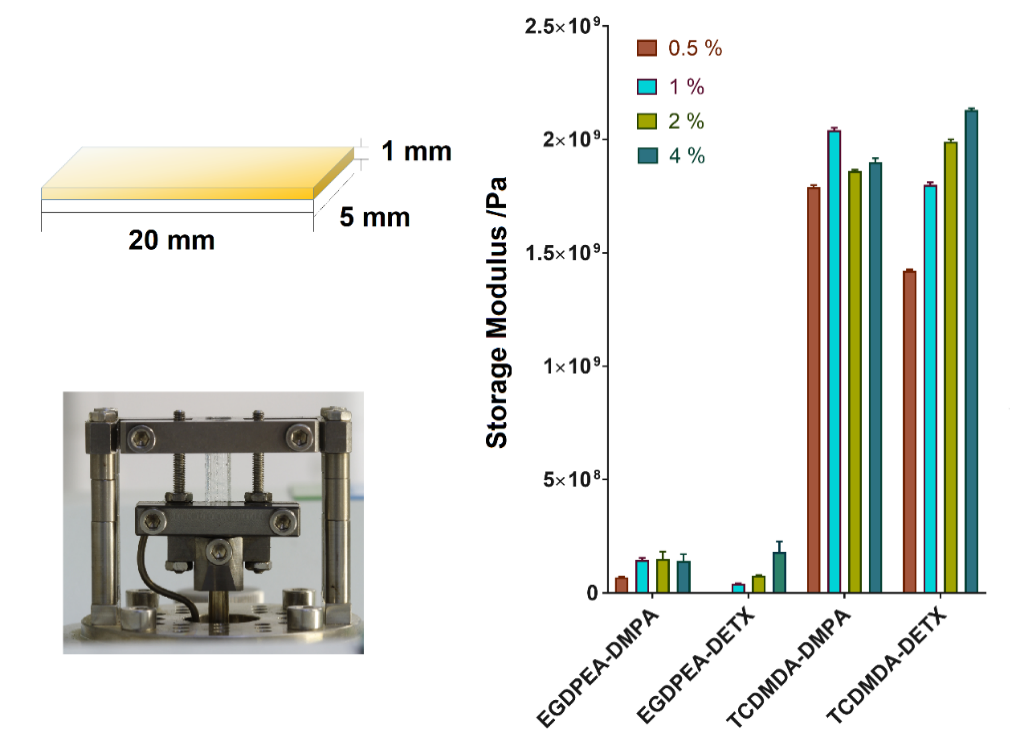


Figure S1: The storage modulus of specimens made from all the formulations were measured by dynamic mechanical analysis using strip samples printed (5 mm x 20 mm x 1mm (w x l x h)) (Mean ± Standard Deviation, n = 5).


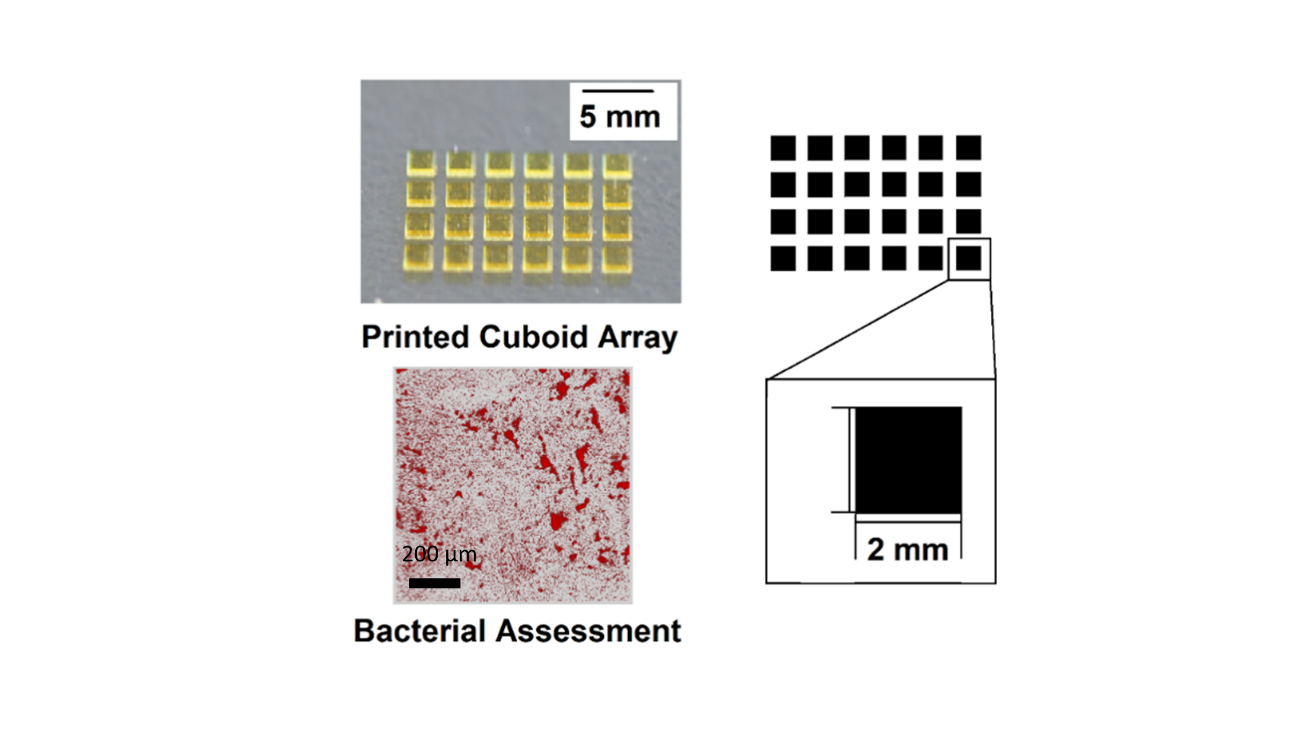


Figure S2: For each formulation, an array of 24 cuboids (2 mm x 2 mm x 0.1 mm; w x l x h) was printed onto polystyrene slides and bacterial biofilm formation tests carried out.


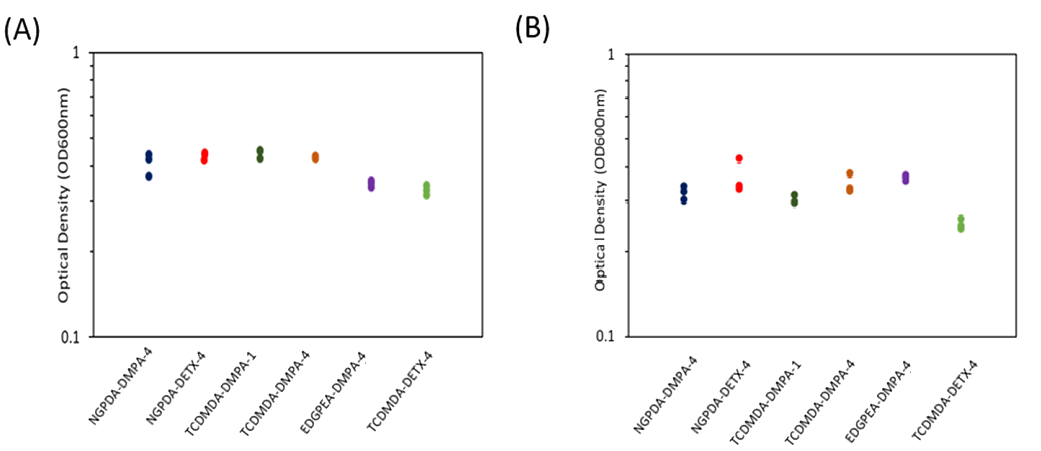


Figure S3: Devices printed with the ink formulations NGPDA-DMPA-4, NGPDA-DETX-4, EDGPEA-DMPA-4, TCDMDA-DMPA-1, TCDMDA-DMPA-4 and TCDMDA-DETX-4 do not inhibit bacterial growth. Samples were immersed in RPMI-1640 medium inoculated with *P. aeruginosa* (**A**) or *S. aureus* (**B**) cells. The stationary phase OD_600_ reached for *P. aeruginosa* and *S. aureus* are shown in (**A**) and (**B**) respectively. Mean ± Standard Deviation, n = 3.


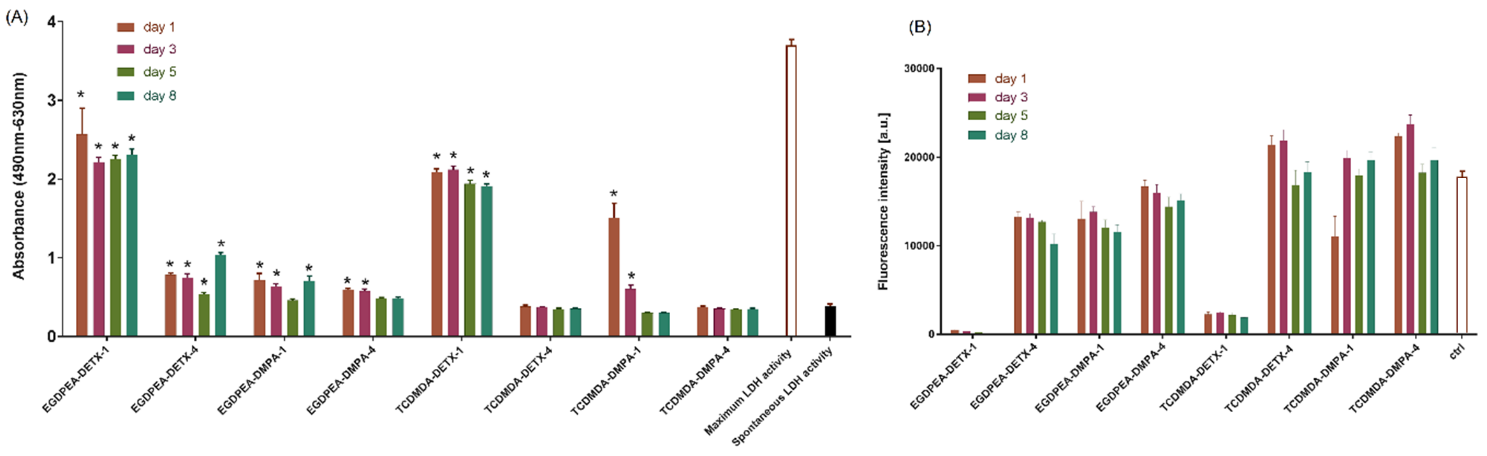


Figure S4: Mammalian cytotoxicity assays using the Presto Blue® assay for printed TCDMDA and EGDPEA samples with both DMPA and DETX initiators at 1 wt % and 4 wt %. The sampling times were 1, 3, 5 and 8 days. The data presented are mean ± standard deviation, n=5 (*p ≤ 0.05).

*
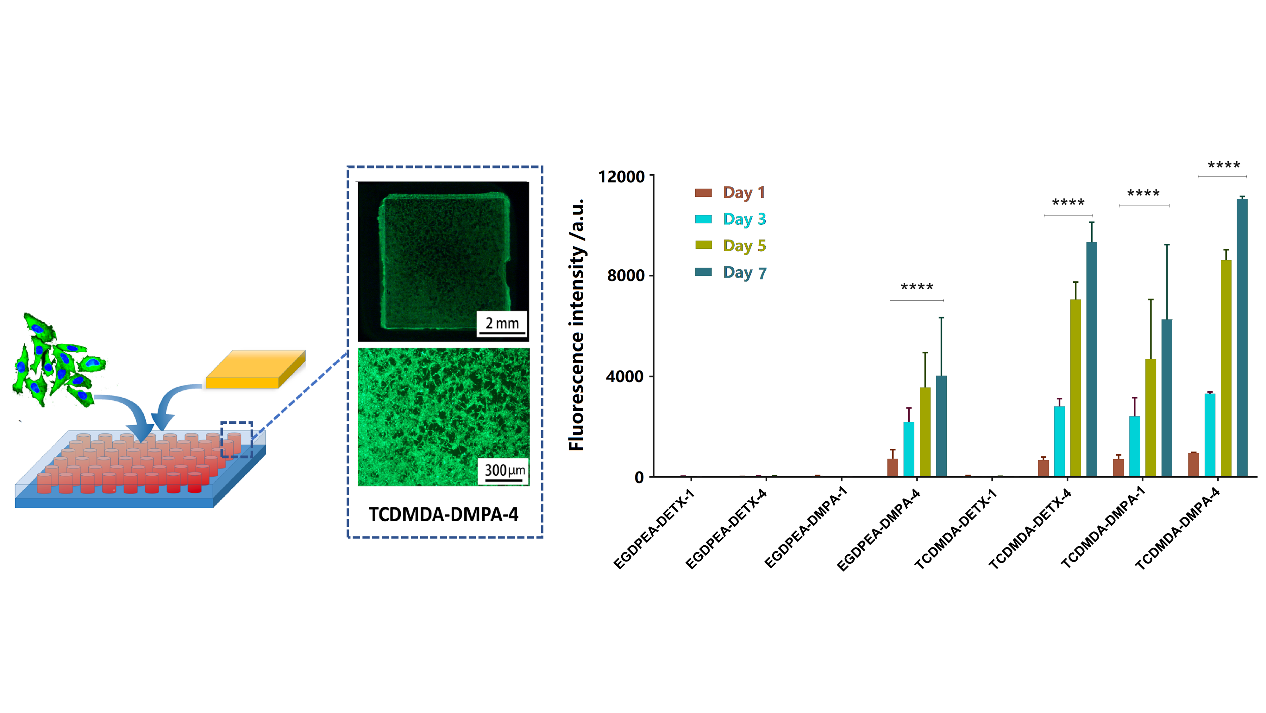
*

Figure S5: Fluorescence intensity of 3T3 cells seeded on the samples in different formulations measured using the Presto Blue assay. The cells adhered and proliferated on 4 formulations (EGDPEA-DMPA-4, TCDMDA-DETX-4, TCDMDA-DMPA-1, and TCDMDA-DMPA-4); cells cultured on TCDMDA-DMPA-4 demonstrated the highest cell metabolic activities at day 7. Mean ± standard deviation, n = 5 (*p ≤ 0.05).


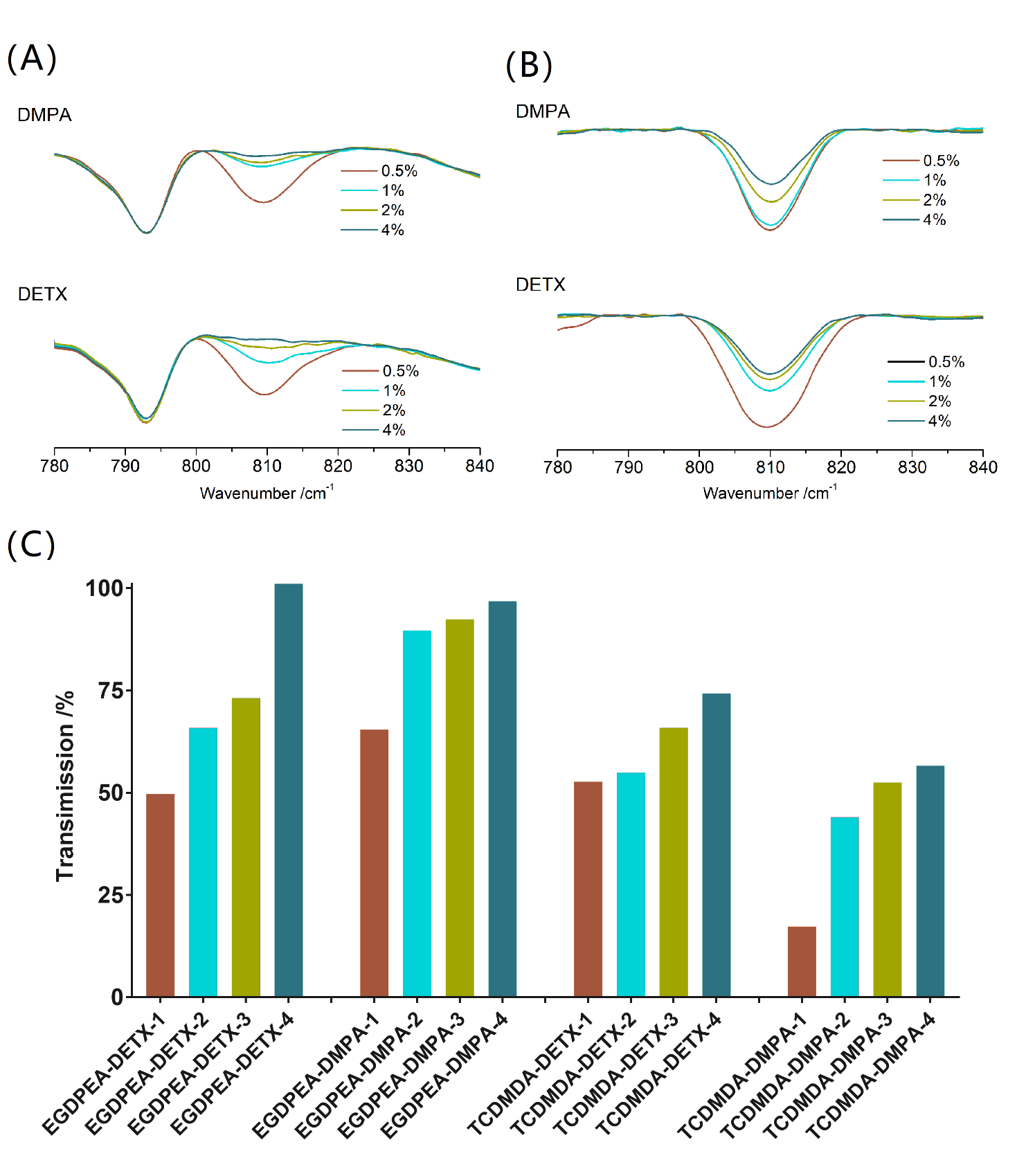


Figure S6: ATR-IR analysis highlighting the trends in residual alkene groups representing the residual monomer and inversely proportional to the level of conversion. The 810 cm-1 absorption seen in the transmission spectrum assigned to the C-H out-of-plane bending vibration of the alkene group displayed normalised to the background intensity. The materials interrogated were A) poly-EGDPEA, and B) poly-TCDMDA; C) Plot of the transmission peak height at 810 cm-1.

*Correlation Analysis*

Statistical analysis was performed using GraphPad Prism 6: Pearson’s correlation coefficient was introduced to quantify the correlation of the residual C=C groups versus bacterial surface coverage, mammalian cell cytotoxicity and storage modulus of the printed polymeric structure.

$$r=\frac{\sum_{i=1}^{n} (x_{i}-\bar{x})(y_{i}-\bar{y})}{\sqrt{{\sum_{i=1}^{n} (x_{i}-\bar{x})}^{2}}\sqrt{{\sum_{i=1}^{n} (y_{i}-\bar{y})}^{2}}}$$

where $n$ is the sample size, $x_{i}$ and $y_{i}$ are the single data points, $\bar{x}$ and $\bar{y}$ are the mean value.


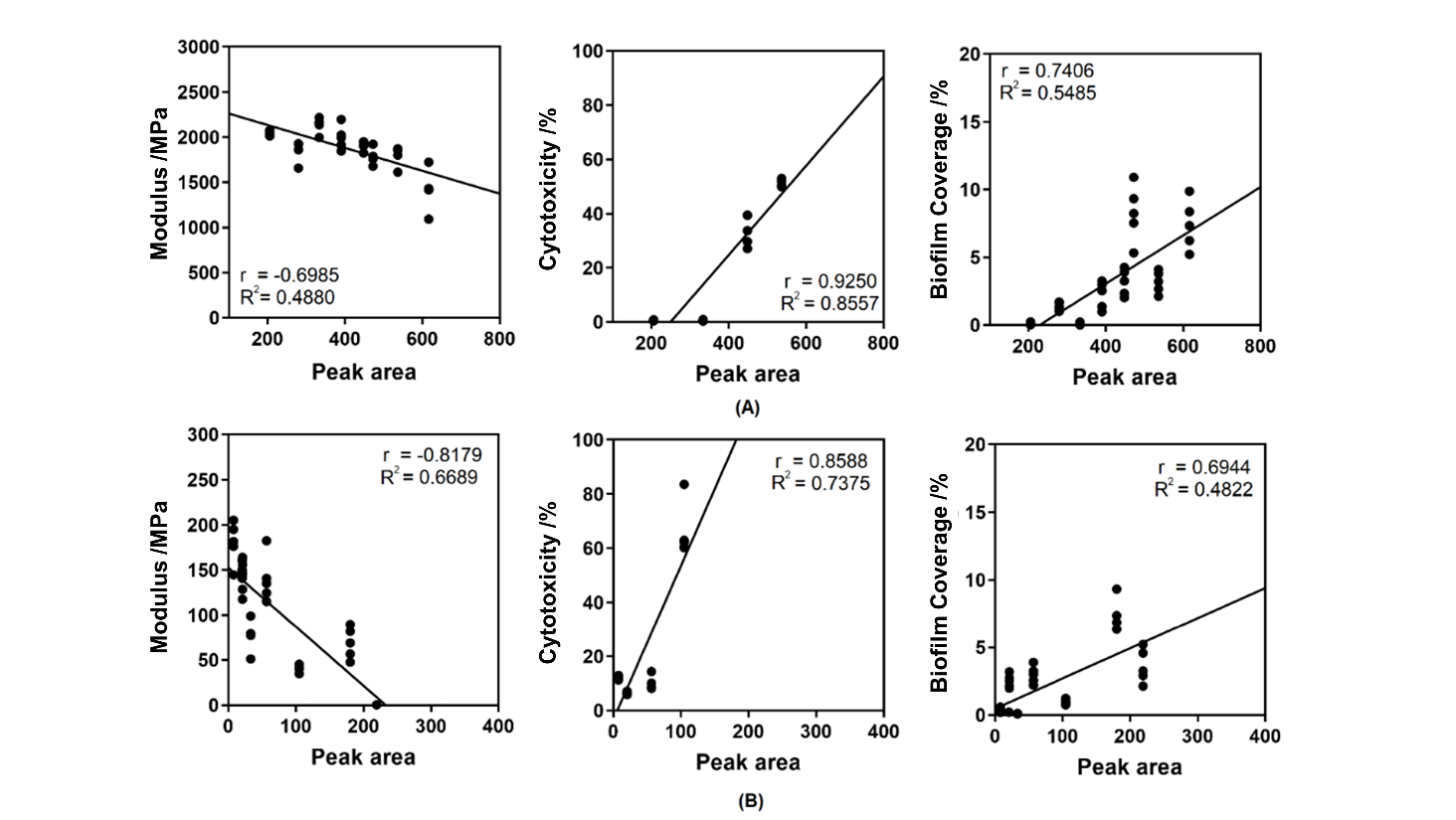


Figure S7: Pearson’s correlation analysis between the C=C residual level (peak height) and bacterial biofilm formation, cytotoxicity and storage modulus respectively. The residual monomer level was judged by the peak area of C-H out-of-plate bending vibration on C=C at 810 cm^-1^. (A) EGDPEA. (B) TCDMDA

­­
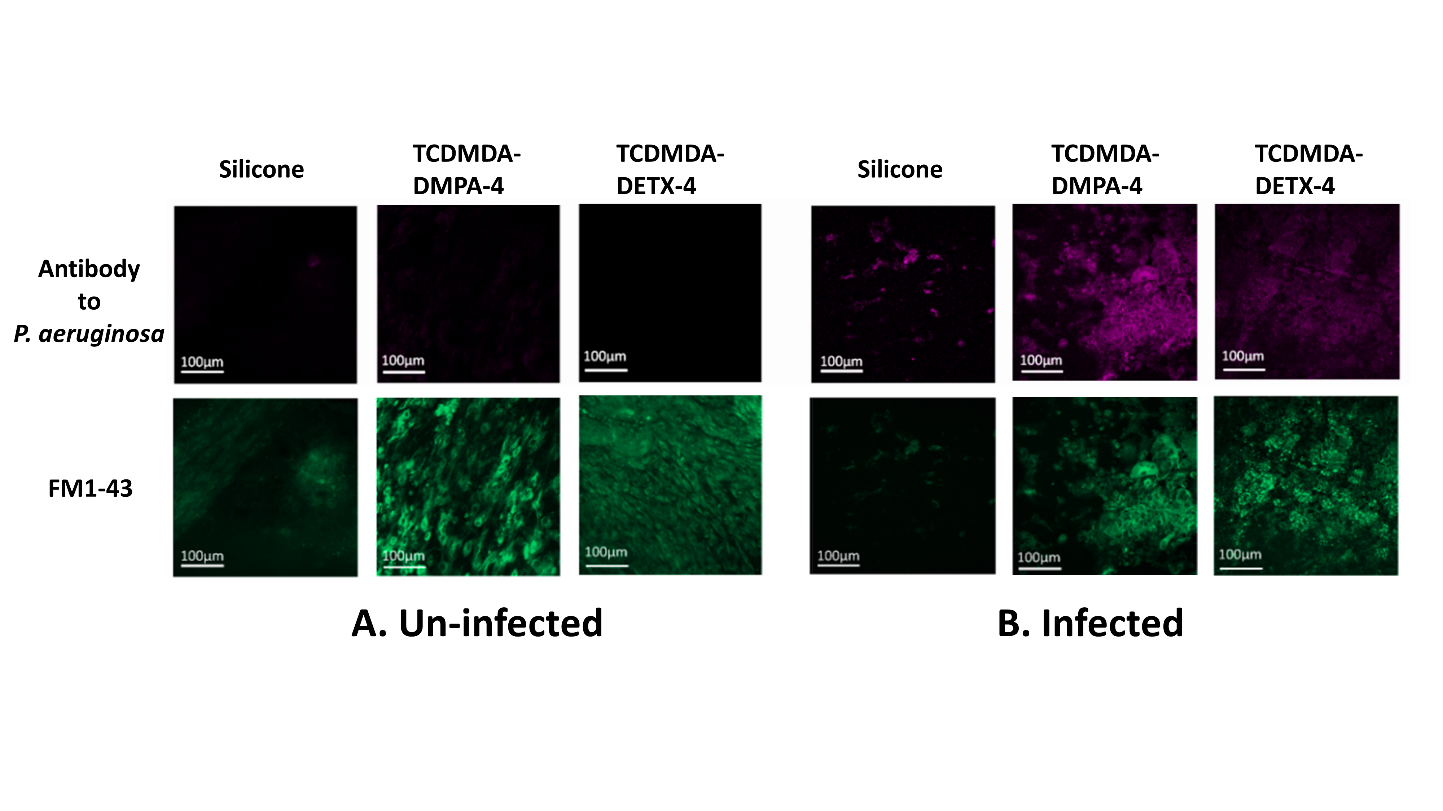


Figure S8. Immunohistochemistry of the TCDMDA and silicone implants *ex vivo* showing the presence of bacteria and host cells. TCDMDA and silicone implants were recovered from (**A**) control, uninfected mice and (**B**) mice infected with *P. aeruginosa*. Implants were stained with an antibody to *P. aeruginosa* (magenta) and with the membrane stain FM1-43 (green). No bacteria were detected on the uninfected controls. On silicone, many individual whole bacterial cells and some bacterial aggregates are apparent. For both TCDMDA formulations there is a stronger host response than for the silicone control and material reactive with the *P. aeruginosa* antibodies was associated with host cells indicative of internalized bacterial cells and their fragments. The conclusion that the cells are not viable, is reached by combining this *P. aeruginosa* antibody data with the lack of luminescence signal from the *ex vivo* implants seen in Figure 3D.


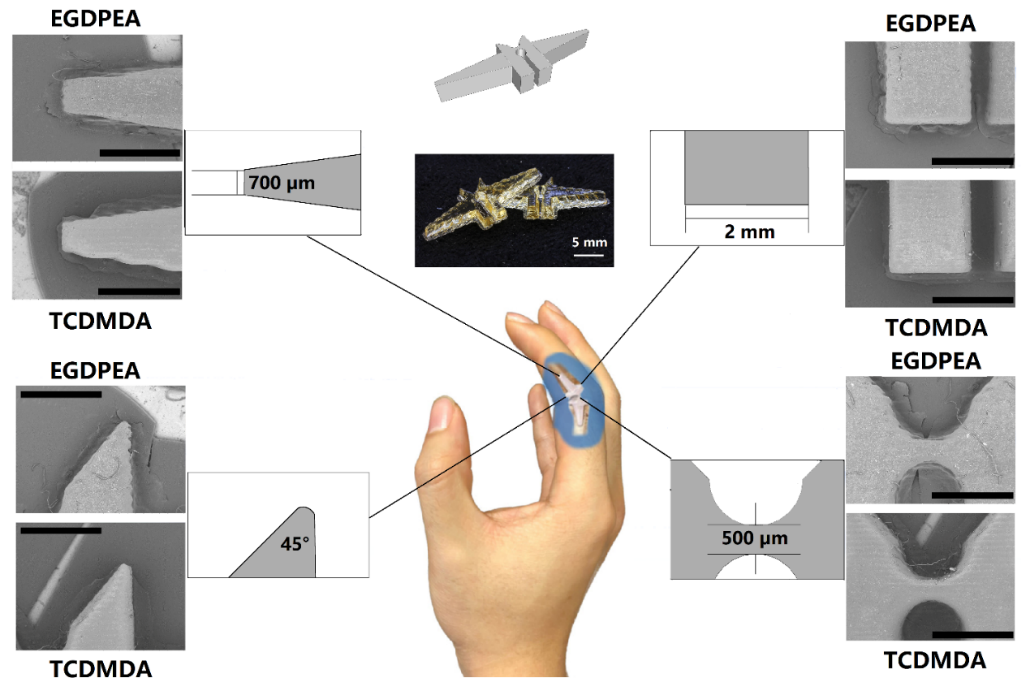


Figure S9: ink-jet based 3D-printing printed finger prosthesis with the developed ink formulations, composed of a central hinge region between two stems. The material formulations used were poly-TCDMDA-DMPA-4 based and poly-EGDPEA-DMPA-4 based, scale bars in the SEM images are 2 mm.


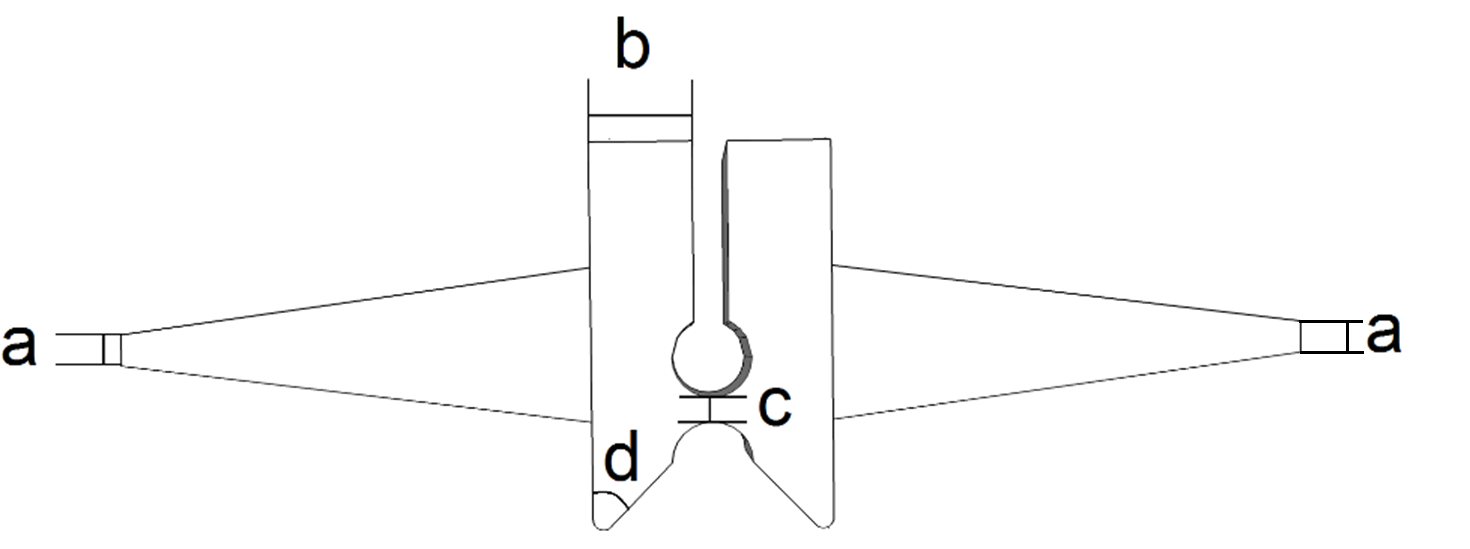


|  | Design | EGPEDA | TCDMDA |
| --- | --- | --- | --- |
| a | 700 µm | 741 µm | 710 µm |
| b | 2000 µm | 2104 µm | 2054 µm |
| c | 500 µm | 544 µm | 507 µm |
| d | 45° | 42° | 44° |

Figure S10: Comparision of the CAD designed feature size with the actual printed specimen size from 4 different key feature points (a-d).


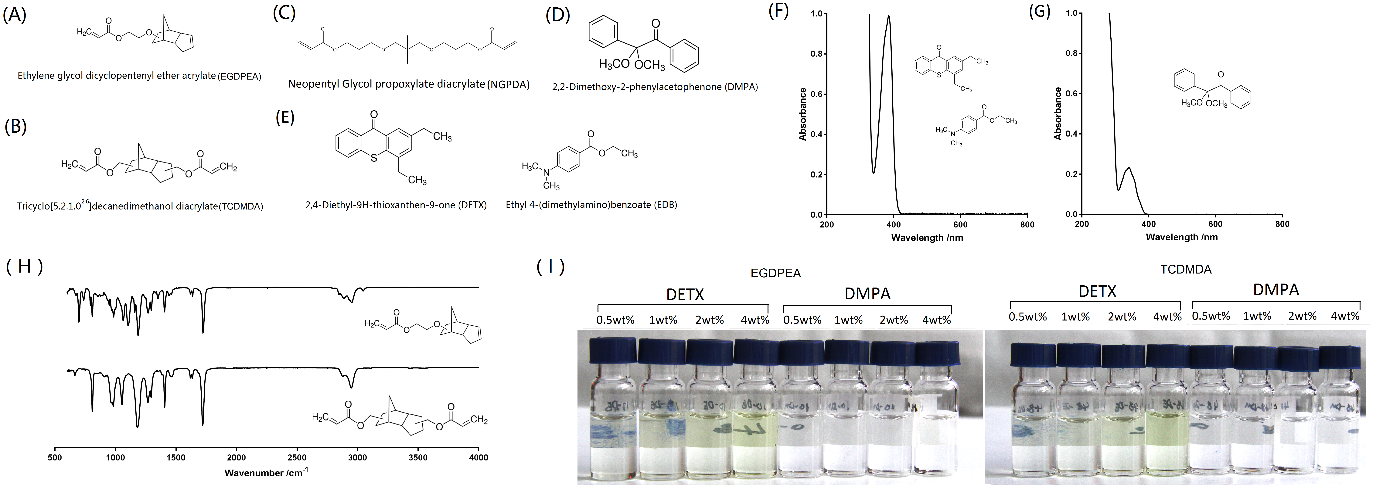


Figure S11: (A-C). Three monomers were selected on the basis of their previously published performance with respect to bacterial biofilm formation^[24-26]^; (D-E) Two different photoinitiators systems, a Type I (D) and a Type II (E) were used for different printing environments; (F-G) UV-VIS absorbance spectra for the photoinitiators; (H) IR spectrum of the monomers before curing. I) Appearance of the ink formulations with different photoinitiators. For DMPA, a clear formulation was obtained, whereas inks with DETX were yellowish.
